# German translation and external validation of the Radboud Skills Questionnaire in patients suffering from Complex Regional Pain Syndrome 1

**DOI:** 10.1186/1471-2474-11-107

**Published:** 2010-06-01

**Authors:** Florian Brunner, Carolin Heitz, Rudolf Kissling, Alfons GH Kessels, Roberto SGM Perez, Johan Marinus, Gerben ter Riet, Lucas M Bachmann

**Affiliations:** 1 Department of Physical Medicine and Rheumatology, Balgrist University Hospital, Zurich, Switzerland; 2 Department Physiotherapy, Balgrist University Hospital, Zurich, Switzerland; 3 Clinical Epidemiology and Medical Technology Assessment, University Hospital Maastricht, Maastricht, Netherlands; 4 Department of Anaesthesiology, VU University Medical Center, Amsterdam, Netherlands; 5 TREND (Trauma Related Neuronal Dysfunction) consortium http://www.trendconsortium.nl/home-en; 6 Research Institute for Extramural Medicine (EMGO), VU University Medical Center, Amsterdam, Netherlands; 7 Department of Neurology, Leiden University Medical Center, Netherlands; 8 Department of General Practice, AMC University of Amsterdam, Meibergdreef 15, Amsterdam, Netherlands; 9 Horten Centre for patient oriented research, University of Zurich, Zurich, Switzerland

## Abstract

**Background:**

Patients suffering from Complex Regional Pain Syndrome commonly complain of substantial limitations in their activities of daily living. The Radboud Skills Questionnaire measures alterations in the level of disability of patients with Complex Regional Pain Syndrome, but this instrument is currently not available in German. The goals of our study were to translate the Dutch Radboud Skills Questionnaire into German and to assess its external criterion validity with the German version of the Disabilities of the Arm, Shoulder and Hand Questionnaire.

**Methods:**

We translated the Radboud Skills Questionnaire according to published guidelines. Demographic data and validity were assessed in 57 consecutive patients with Complex Regional Pain Syndrome 1 of the upper extremity. Information on age, duration of symptoms, type of Complex Regional Pain Syndrome 1 and type of initiating event was obtained. We assessed the external criterion validity by comparing the German Radboud Skills Questionnaire and the German Disabilities of the Arm, Shoulder and Hand Questionnaire and calculated the prediction intervals.

**Results:**

Score values ranged from 55.4 ± 22.0 for the Disabilities of the Arm, Shoulder and Hand Questionnaire score and 140.1 ± 39.2 for the Radboud Skills Questionnaire. We found a high correlation between the Radboud Skills Questionnaire and the Disabilities of the Arm, Shoulder and Hand Questionnaire (R^2 ^= 0.83).

**Conclusion:**

This validation of the Radboud Skills Questionnaire demonstrates that this German version is a simple and accurate instrument to assess and quantify disabilities of patients suffering from Complex Regional Pain Syndrome 1 of the upper extremity for clinical and research purposes

## Background

Complex Regional Pain Syndrome (CRPS) is a painful condition that often results in substantial disability [1]. Two types of CRPS can be distinguished: type 1, formerly known as reflex sympathetic dystrophy or algodystrophy, which occurs without a definable nerve lesion and type 2, formerly called causalgia, in which a definable nerve lesion is present [2]

Most of the measurement instruments that assess the condition of patients suffering from CRPS 1 focus on impairments on a structural and functional level [3-8]. So far, activity limitations have not been evaluated as extensively as the impairments caused by CRPS. As a consequence, little information is available on the problems CRPS patients encounter in activities of daily living.

One global instrument to assess symptoms and functional status of the upper extremity is the Disabilities of the Arm, Shoulder and Hand Questionnaire (DASH) [9]. The DASH has been validated in German [10] and is widely used in clinical practice and research to measure activity and limitations in patients with upper extremity musculoskeletal conditions including CRPS [10]. One of the advantages of the self-administered DASH is that it includes symptoms and disabilities of the whole upper extremity and that it can be applied in a broad range of disorders. However, methodological work has shown that disease-specific questionnaires are generally more responsive to change and that they are more relevant to patients than global instruments [11].

The Radboud Skills Questionnaire (RASQ) [12] is a valid instrument for measuring alterations in the level of functional tasks of the upper extremity that has been developed specifically for patients with CRPS. The original version of this questionnaire is in Dutch. Our aim was to translate the RASQ into German and to verify its external validity by comparing it with the German version of the DASH (DASH_G_).

## Methods

### Recruitment sources and data acquisition

We recruited patients from the outpatient clinic of Balgrist University Hospital, Zurich, Switzerland and through advertisements posted on two self-help homepages for patients afflicted with CRPS 1 (http://www.morbus-sudeck.ch, http://sudeck.foren-city.de). We included all eligible and consenting adult patients suffering from CRPS 1 of the upper extremity with fulfilled International Association for the Study of Pain (IASP) criteria [13], more than 18 years of age, illness duration of more than three months and the ability to complete the questionnaires. The study protocol was approved by the local Ethics Committee (Spezialisierte Unterkomission für Orthopädie der Kantonalen Ethikkommission, Zurich, Switzerland) and informed consent was obtained from all participants.

### Assessment instruments

In 1999, Oerlemans et al. constructed and validated the RASQ to measure the level of disability in activities of daily living in patients with CRPS 1 of the upper extremity [12].

The questionnaire construction was based on the Dutch elaboration of the International Classification of Impairments, Disabilities and Handicaps (ICIDH), an earlier version of the International Classification of Functioning, Disability and Health (ICF) model [14]. The questionnaire contains items of 'disabilities due to hand disease' domain of the ICF model. In order to be applied to both hands, the questionnaire includes items referring to two-handed activities in daily living or social activities. The constructed questionnaire was judged for its merit in Delphi rounds with experts and then readjusted. The original version of the RASQ questionnaire consists of 11 categories, with the first four addressing personal care (15 questions), the next three addressing domestic activities (17 questions) and the latter four addressing other activities (13 questions). The RASQ was reliable in terms of response stability (median coefficients of variation 2.2 to 6.6%) and the correlations between categories of items were fair to good [12]. A numeric score (1-5, with an extra score of '9' for 'not applicable' for patients who never perform the involved activity) is assigned to each question of the RASQ. In order to avoid implications on the scores, we scored every missing or not applicable response with the mean score of the specific question. The Disabilities of the Arm, Shoulder and Hand Questionnaire (DASH) questionnaire is a standardized patient-completed upper extremity outcome measure [9]. Using a self-reporting system, patients choose scores of on to five to 30 items relating to impairments and activity limitations, as well as participation restrictions in leisure activities and work. The DASH questionnaire was translated in many languages. The German questionnaire was obtained from http://www.dash.iwh.on.ca.

### Translation process

**Figure 1:**
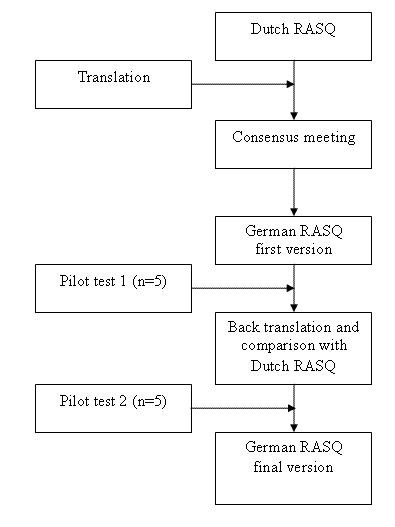
**Flow diagram of the development process of the German RASQ**.

We followed a sequential forward and backward translation approach (see figure 1) [15]. Two professional translators translated the original Dutch version of the RASQ into German. In a consensus meeting a rheumatologist, a specialist in physical medicine and rehabilitation, a physical therapist and an epidemiologist assessed the consistency of the translation and judged its face validity. They then agreed on the first German version for these formats. The questionnaire was pilot tested in five CRPS 1 patients to identify difficulties in comprehension and interpretation of the questions. In addition, we tested various possible wordings of items, answer choices and instructions if the translation team considered more than one possible version. A Dutch translator with experience in biomedical sciences but unaware of the original versions performed a back translation of the German version into the source language (Dutch). A team of experts (a rehabilitation specialist, a rheumatologist, an epidemiologist and a physical therapist) compared the back translation with the Dutch versions to check for conceptual discrepancies. After a second pilot test (n = 5 CRPS 1 patients), the translation team discussed the comments from these patients and decided by consensus on modifications. Finally, the experts approved the final German version of the RASQ (RASQ_G_).

### Validity assessments

Both questionnaires were offered to CRPS 1 patients meeting the inclusion criteria. Based on formal sample size calculations for regression analysis the required number of patients for the validity assessment was established at 50. Patients received both questionnaires either during a visit to our outpatient clinic or by mail. Participants were asked to complete both questionnaires on the same day and to mail them back to our institution.

### Statistical analysis

Values are reported as mean ± SD, medians and interquartile ranges (IQR) or as absolute number and percentage. Linear regression analysis and individual prediction intervals were used to assess the relationship between the RASQ_G _and the German DASH questionnaire (DASH_G_). Data storage and statistical analyses were performed with the SPSS 12 statistical software package (SPSS Inc. Headquarters, 233 S. Wacker Drive, 11th floor Chicago, Illinois 60606).

## Results

### Translation and instrument development

The wording of the questions and response options correspond to the original version. We did not add or remove items nor changed the response categories.

### Demographic and clinical characteristics

The demographic and clinical characteristics of the participants are shown in table 1. We enrolled 57 patients suffering from CRPS 1 of the upper extremity in this study (female/male 37/20). Surgery was the most common initiating event (54.4%). Median disease duration was 1.9 years (IQR 0.9 to 4.3). Average score for the RASQ_G _was 140.1 ± 39.2 and for the DASH_G _55.4 ± 22.0.

**Table 1:** Characteristics of study population (N = 57)

Characteristic	Variable
Gender	
Male	20 (35.1%)
Female	37 (64.9%)
Mean age (± standard deviation) Age range	53.4 ± 11.8 years 21.2-81.8 years
Median number of years with CRPS 1	1.9 years (0.9 to 4.3)
Initiating event	
Trauma	21 (36.8%)
Surgery	31 (54.4%)
Other	5 (8.8%)
RASQ_German_^+^	140.1 ± 39.2
DASH_German_"	55.4 ± 22.0

^+^RASQ_German_: German version of Radboud Skills Questionnaire

^"^DASH_German_: German version of Disabilities of Shoulder, Arm and Hand Questionnaire

### High correlation between the RASQ_G _and the DASH_G_

**Figure 2:**
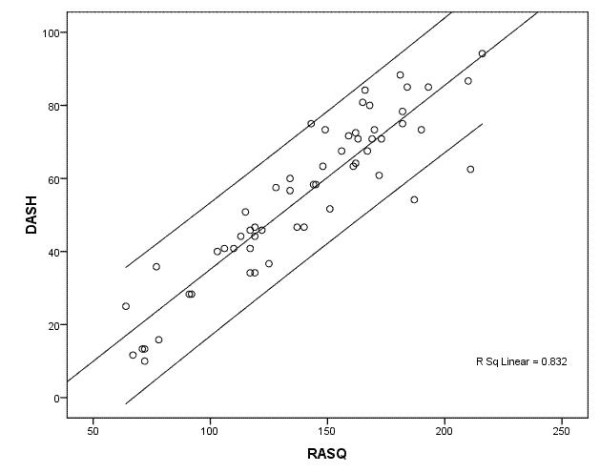
**Linear regression line with 95% prediction intervals for an individual**.

We found a high correlation between the RASQ_G _and the DASH_G _(R^2 ^= 0.83). The regression function showed an intercept of -15.2 and a slope of 0.50. Figure 2 shows the 95% prediction intervals and the regression line.

## Discussion

To our knowledge, this is the first German translation and external validation of the originally Dutch version of the RASQ, allowing the standardized measurement of activity limitations of patients suffering from CRPS 1 of the upper extremity. We found that the disease-specific German RASQ (RASQ_G_) had excellent correlation with the global German DASH (DASH_G_) questionnaire. Given its higher theoretical potential to detect changes in disability (responsiveness) the disease-specific RASQ arguably should be the instrument of choice when assessing patients with CRPS 1 of the upper extremity.

The translation process itself had no issues of concern, all forward and backward translations were consistent with each other and with the original version. We followed the rigorous translation method proposed by Wild et al. [16], which consisted of a forward and backward translation by professional translators, and by a consensus meeting between researchers. By applying this robust methodology we ensured that the content, integrity and essence of the RASQ items are maintained and expressed clearly and accurately from one language to another.

Our study has several limitations. First, since diagnosis of CRPS 1 is still a matter of debate our sample might not be representative for a larger CRPS 1 population. The diagnosis of CRPS 1 is based on clinical findings (including sensory, autonomic, motor and trophic changes) and the fulfilment of established diagnostic criteria [17]. We only included patients fulfilling the criteria established by the International Association for the Study of Pain (IASP) [13] in all participants. However, these IASP criteria have been criticized because they are symptom based and show a low specificity [18]. Second, the RASQ is an instrument characterized by measuring functioning as perceived and recalled by the participants. In a study by Schasfoort et al. the authors showed that activity of the upper extremity measured by an upper limb activity monitor only had a weak or non-specific relationship with the RASQ and other similar instruments, including the DASH [19]. This indicates that the actual activity potentially differs from measured perceived functioning in questionnaires. However, we do not think that this is an actual limitation of the study, since it has been shown that the correlation between impairment and disability is generally only weak to moderate [20].

This validated German version of the RASQ will help determine disability in patients suffering from CRPS 1 of the upper extremity in German speaking countries and is appropriate for clinical practice as well as research. In addition, it allows for a comparison of the results of studies from different countries. In particular, the German version of the RASQ allows us now to collect data for the Swiss CRPS 1 cohort study [21] and to compare the results with those of our Dutch collaborator in the TREND consortium (Trauma RElated Neuronal Dysfunction, http://www.trendconsortium.nl). Investigators in German-speaking countries now have the possibility to assess physical activity and limitations in daily living in patients with CRPS 1 of the upper extremity using the German RASQ. However, other psychometric qualities such as reliability and responsiveness have to be studied in future projects [22].

## Conclusions

In conclusion, the assessment of disability is essential for the management of patients suffering from CRPS 1. Disability may negatively interact with performance levels and outcome measures, and therefore needs to be taken into consideration when caring for these patients. This validation of the RASQ demonstrates that this German version is a simple and valid instrument to assess and quantify disability of patients suffering from CRPS 1 of the upper extremity for clinical and research purposes.

## Competing interests

The authors declare that they have no competing interests.

## Authors' contributions

All authors participated in the study design. FB and LMB drafted the protocol and the manuscript. CH assisted in patient recruiting. RK and FB obtained funding. AGHK, RSGMP, JM and GR critically reviewed the protocol and the manuscript.

## Pre-publication history

The pre-publication history for this paper can be accessed here:

http://www.biomedcentral.com/1471-2474/11/107/prepub
